# Open-source algorithm and software for computed tomography-based virtual pancreatoscopy and other applications

**DOI:** 10.1186/s42492-022-00116-1

**Published:** 2022-08-03

**Authors:** Haofan Huang, Xiaxia Yu, Mu Tian, Weizhen He, Shawn Xiang Li, Zhengrong Liang, Yi Gao

**Affiliations:** 1 School of Biomedical Engineering, Health Science Center, Shenzhen University, Shenzhen, 518060 China; 2 Laboratory for Imaging Research and Informatics, State University of New York, Stony Brook, NY 11794 USA; 3 Shenzhen Key Laboratory of Precision Medicine for Hematological Malignancies, Shenzhen, 518060 China; 4 Marshall Laboratory of Biomedical Engineering, Shenzhen, 518060 China; 5 Peng Cheng Laboratory, Shenzhen, 518066 China

**Keywords:** Pancreatic cancer, Pancreatic duct segmentation, Virtual pancreatoscopy, 3D Slicer

## Abstract

Pancreatoscopy plays a significant role in the diagnosis and treatment of pancreatic diseases. However, the risk of pancreatoscopy is remarkably greater than that of other endoscopic procedures, such as gastroscopy and bronchoscopy, owing to its severe invasiveness. In comparison, virtual pancreatoscopy (VP) has shown notable advantages. However, because of the low resolution of current computed tomography (CT) technology and the small diameter of the pancreatic duct, VP has limited clinical use. In this study, an optimal path algorithm and super-resolution technique are investigated for the development of an open-source software platform for VP based on 3D Slicer. The proposed segmentation of the pancreatic duct from the abdominal CT images reached an average Dice coefficient of 0.85 with a standard deviation of 0.04. Owing to the excellent segmentation performance, a fly-through visualization of both the inside and outside of the duct was successfully reconstructed, thereby demonstrating the feasibility of VP. In addition, a quantitative analysis of the wall thickness and topology of the duct provides more insight into pancreatic diseases than a fly-through visualization. The entire VP system developed in this study is available at https://github.com/gaoyi/VirtualEndoscopy.git.

## Introduction

Pancreatic cancer arises when cells in the pancreas, a glandular organ behind the stomach, begin to multiply out of control and form a mass, and is associated with a relatively poor prognosis and high mortality rate. In 2012, pancreatic cancer was the seventh most common cause of cancer-related deaths worldwide, resulting in 330,000 deaths, a rate that is still increasing annually [1, 2]. Pancreatic cancer is typically detected at an advanced stage, and most treatment regimens are ineffective, contributing to a poor overall prognosis [3]. The most common type, pancreatic adenocarcinoma, accounts for approximately 90% of cases, and the term “pancreatic cancer” is sometimes used to refer only to this type.

Intraductal papillary mucinous neoplasms (IPMNs) are pancreatic cystic lesions originating from the intraductal growth of mucin-producing cells [4–6]. Pancreatography provides an excellent visualization of vascular patterns and tumor vessels that harbor malignancy [7, 8], improving the identification of tumor cells. Furthermore, it can provide valuable data for the differential diagnosis of amorphous filling defects in the main pancreatic duct, as well as for assessing the location and extent of nodules/tumors, allowing the best surgical procedure to be selected [6]. Hara et al. [9] classified the appearance of protruding lesions in pancreatoscopy images, achieving an accuracy of 88% in distinguishing malignant from benign IPMNs for the main duct, and 67% for branch ducts. Tyberg et al. [10] reported the first use of digital single-operator cholangiopancreatoscopy for pre-surgical mapping of pancreatobiliary malignancy. Sixty-two percent of patients undergoing surgery to remove an IPMN had a change in their surgical plan based on preoperative pancreatoscopy. In terms of treatment, 28 patients having underwent pancreatoscopy-guided lithotripsy were retrospectively studied by Attwell et al. [11], who reported that Wirsung duct clearance was achieved in 79% of cases, with clinical success achieved in 89% of cases at a median follow-up of 13 mos. Recent studies by Shah et al. [12] and Navaneethan et al. [13] obtained pancreatic duct clearance in 100% (7/7) and 80% (4/5) of patients with lithotripsy (electrohydraulic and laser, respectively) using a digital single-operator cholangiopancreatoscope [14].

Although pancreatoscopy plays a significant role in both the diagnosis and treatment of pancreatic diseases, there are several limitations. First, the overall complication rates after diagnosis and therapy are within the range of 10% to 12%, mainly indicating mild pancreatitis [15, 16]. Other possible adverse events include bleeding, perforation, and pancreatitis [17, 18]. In addition, acute pancreatitis may be induced by the passage of the pancreatoscope or excessive intraductal irrigation, which is required for improved visualization [6].

The success of pancreatoscopy can depend on the anatomy and diameter of the main pancreatic duct, ductal stenosis, or blocking stones. Depending on the clinical indication, the visualization rate of the Wirsung duct is only 70% to 80%. A main pancreatic duct with a diameter of greater than 5 mm is essential for a successful pancreatoscopy [14].

Access to the pancreatic duct is similar to that of mother-baby cholangioscopy and commonly occurs through the major papilla, with or without sphincterotomy, depending on the diameter of the pancreatic orifice and diagnostic indications, as well as through the minor papilla [19]. However, it is important to determine whether sphincterotomy should be performed prior to pancreatic duct cannulation, which may be extremely helpful in cases with strictures and complex biliary stones. However, pancreatoscopy is costly because it requires a second light source and a powerful processor for the baby scope. In addition, the use of a reusable baby scope requires significant maintenance costs owing to its fragility [6].

Advances in computer technology have led to the development of novel medical-imaging techniques. Virtual endoscopy (VE) is a non-invasive technique that amplifies the perception of cross-sectional images acquired through axial computed tomography (CT) in a 3D space, providing precise spatial relationships between pathological regions and their surrounding structures [20]. In general, VE can provide information on many hollow anatomical structures and has already been used for the exploration of the trachea, colon, aorta, brain ventricles, nasal cavity, and paranasal sinuses [20].

CT-based virtual colonoscopy (VC), that is, CT colonography (CTC), has shown a performance comparable to that of optical colonoscopy (OC) in the detection of polyps of 8 mm and larger in a less invasive and more cost-effective manner. Magnetic resonance imaging-based VC, that is, MR colonography, has certain advantages over CTC in terms of differentiating the polyps from other tissues and colonic materials; however, its lower spatial resolution and proneness to motion artifacts are two main drawbacks in comparison with CTC [21, 22]. Several scholars have proposed an easily accessible virtual bronchoscopy system for navigation in the lung, assisting the user with a complete set of tools that facilitate navigation toward user-selected regions of interest [23]. Sata et al. [24] assessed the application of CT-virtual pancreatoscopy (CT-VP) created using multidetector row CT in the clinical diagnosis of an IPMN in the pancreas. The authors found that CT-VP and 3D-CT pancreatographic images are finer in quality and that the procedures are less invasive, faster, and less expensive. Another study pointed out that virtual bronchoscopy simulations accurately represent findings confirmed through real bronchoscopy [25].

In addition, Nain et al. [26] designed a 3D VE system to facilitate the diagnostic and surgical planning phases of endoscopic procedures. This system allows the user to interactively explore the internal surface of a 3D patient-specific anatomical model and to create and update a fly-through trajectory using the model in an endoscopic simulation.

In summary, there have been relatively few studies conducted on VP. Instead, researchers have paid more attention to pancreatic segmentation. However, because the pancreatic duct is smaller and has lower contrast than other tubular structures such as the esophagus and bronchus, segmentation of the pancreatic duct remains a challenge [27]. Sata et al. [24], Nakagohri et al. [28] and Tanizawa et al. [29] investigated the usefulness of VP for achieving a diagnosis but provided no details about the algorithm, let alone the open-source code applied.

In this study, a super-resolution segmentation scheme is presented that can extract the pancreatic duct from conventional CT/MR images. The scheme uses an optimal path algorithm to identify the centerline of the duct and super-resolution segmentation is then applied to extract the lumen. Algorithms on the optimal path described in the existing literature are often used for other tubular structures, such as coronary artery extraction in contrast-enhanced CTA images. There are two main types of methods found in the literature: semi-automatic and fully automatic. With a semi-automatic method, a manual labeling approach similar to that used in this study is adopted, and the algorithm extracts the optimal curve among the marked points. With an automatic method, by contrast, the centerline of the coronary artery is extracted using techniques such as a Hough transform [30], particle filter [31], neural network [32, 33], or distance transformation [27]. For more prominent tubular structures such as coronary arteries, the method of extracting the centerlines from CTA images has been proven to be an accurate and effective approach.

However, when applying VP, because the resolution and contrast around the pancreatic duct are extremely low, the duct is inconspicuous in the image. Although some studies have used methods based on a distance transformation-modulated convolutional neural network to directly extract the pancreatic duct without extracting the centerline first [27], according to the results reported, the accuracy remains unsatisfactory. Thin-slice abdominal CT scans have a slice thickness of approximately 1 mm. At such a resolution, the pancreatic duct occupies only 3–5 pixels. Consequently, although the human eye can see the trace of the duct, when using the computational method for extraction and segmentation, a consistent and correct geometric structure cannot be obtained at the original resolution. This type of phenomenon occurs when the scale of the target is close to the pixel resolution and has also been reported under other image segmentation scenarios [34]. Super-resolution methods have also been successfully used to extract fine structures on dense grids that have already been captured visually but cannot be segmented at the original resolution.

Under other scenarios, super-resolution methods are often used to obtain higher-quality images for doctors to view, and the goal of a super-resolution reconstruction is often to obtain images that are similar to those of more advanced imaging equipment. In contrast, this work focuses on using the reconstruction to segment the geometric structure of the target, for which much simpler sub-pixel methods can be used.

Combining these approaches for other organs and structures, a method is presented that incorporates the optimal path and super-resolution segmentation for pancreatic duct extraction. In addition, the proposed algorithm was developed as an interactive module for use in the well-known 3D Slicer framework for clinical assistance.

## Methods

**Fig. 1:**
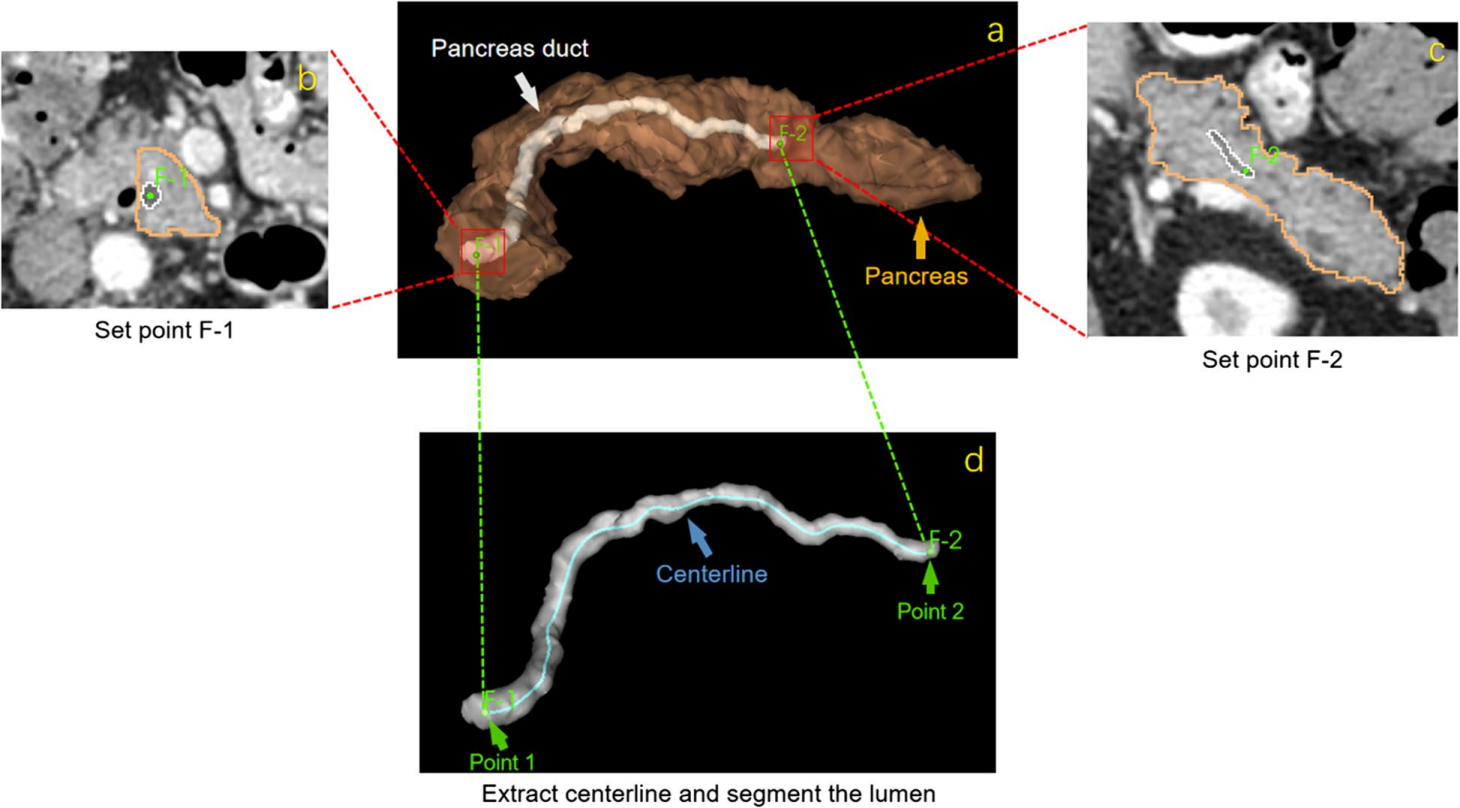
Algorithm diagram: **a** 3D model of the pancreas. Two points (F-1 and F-2) are first set at the **b** head and **c** tail of the duct, respectively. **d** The centerline is then automatically calculated and lumen segmentation is applied

There are two fundamental building blocks for an end-to-end VE framework. First, the centerline of the pancreatic duct must be extracted, based upon which, the category of each pixel around the line is iteratively classified and refined for lumen segmentation. Figure 1 illustrates this process. The user only needs to provide two points to mark the beginning and end of the pancreatic duct. Then, as shown in the bottom part of Fig. 1, both the centerline and the entire duct will be extracted through the algorithm detailed below.

### Centerline extraction of pancreatic duct stones

The CT image to be processed is denoted as $I :\Omega \to {\mathbb{R}}$ where $\Omega \subset {\mathbb{R}}^{3}$. To enhance the vessel regions, the vessel enhancement algorithm, as detailed in ref. [35], is employed. This algorithm is often used as a preprocessing step for vessel segmentation in medical images to improve the overall segmentation accuracy. First, the user is asked to click on a few points $({P}_{i}\in {\mathbb{R}}^{3},i=\mathrm{0,1},\dots ,N-1)$ in the image to mark the initial estimation of the centerline of the vessel. Among these points, the first and last points, ${P}_{0}$ and ${P}_{N-1}$ should be located at the beginning and end of the main pancreatic duct, respectively. Second, an optimal curve $C :[\mathrm{0,1}]\to {\mathbb{R}}^{3}$ is derived that passes through all points. Denote the image of the vesselness as$V :\Omega \to {\mathbb{R}}$. This is then formulated as a constrained optimization problem:

1 $$C=\mathrm{argmin}{\int }_{0}^{1}\left(1-V\left(C\left(t\right)\right)\right)\left|{C}^{t}\left(t\right)\right|dt$$

$$\mathrm{where }C\left(0\right)={P}_{0},C\left(1\right)={P}_{N-1},\mathrm{and}{ P}_{i}\in C,$$

for which ${C}^{t}(t)$ is the tangent vector of the curve $C(t)$.

According to Bellman’s principle of optimality, an optimal policy has a prior property in which regardless of the initial state and initial decision, the remaining decisions must constitute an optimal policy with regard to the state resulting from the first decision. The aforementioned problem can be formulated as follows:

2 $${C}_{i}=\mathrm{argmin}{\int }_{0}^{1}\left(1-V\left({C}_{i}\left(t\right)\right)\right)\left|{C}_{i}^{t}\left(t\right)\right|dt$$

$${C}_{i} :\left[\mathrm{0,1}\right]\to {\mathbb{R}}^{3},$$

$$\mathrm{where}\;C_i\left[0\right]=P_i,C_i\left[1\right]=P_{i+1},i=0,\dots,\mathrm{and}N-2.$$

The tangent vectors are approximated in a discrete fashion using the finite-difference method. Moreover, for piecewise smooth curves, because the optimal curves are computed in a section-by-section manner, the tangent vector is not computed at the joint between two consecutive points. Therefore, after solving each optimal fragment ${C}_{i}$ from Eq. (2), the optimal curve $C$ in Eq. (1) can be obtained as the extracted centerline.

### Lumen segmentation

Once the centerlines are extracted, the vessel lumen is segmented. The pancreas is surrounded by an extremely thin connective tissue capsule that invaginates the gland to form septae, which serve as scaffolding for the large blood vessels. Note that several segmentation algorithms must create a stable boundary between the target and surrounding tissue. For instance, the sparse field level set method employs a set of linked lists to track the active voxels around the interface [36], requiring at least 12 pixels to represent the surface on both sides of the lumen of the duct. Other methods, such as graph cuts or random Walker methods, treat an image as a graph and minimize certain energy functions.

To extract fine-scale morphological features, the image $I$ is upsampled by a factor of$c$. This is denoted by${I}_{c} :{\Omega }^{s/c}\to {\mathbb{R}}$, where the domain ${\Omega }^{s/c} := \left\{\left({x}_{i},{y}_{j},{z}_{k}\right)\right\}$ has a grid density (resolution) of${s/c={x}_{i+1}-{x}_{i}={y}_{i+1}-{y}_{i}={z}_{i+1}-z}_{i}$. The selection of $c$ is critical for detecting fine-scale morphological features.

Such super-resolution tasks have attracted the attention of many researchers within the field of medical image analysis, among others. The computational load is often extremely high owing to the use of advanced techniques such as a sparse reconstruction and/or deep neural network. However, the purpose of this study was to capture geometric shapes with better precision. Following the ref. [34], for this purpose, $c=5$ was chosen along with a simple convex interpolation kernel.

Magnifying an image by $c=5$ on each axis results in an image that is 125-times larger. Hence, upsampling can be applied only within the vicinity of the duct and only when the centerline of the duct can be extracted.

Once the pancreatic duct region is upsampled, segmentation of the lumen is attempted. To this end, the centerline from the curve representation is first converted into a binary image $K :\Omega \to \mathrm{0,1}$ such that$K\left({C}_{i}\left(t\right)\right)=1$; otherwise, the value is zero. The point set of the centerline is denoted by${S}_{1} :=\{x\in {\mathbb{R}}^{3} :K(x)=1\}$. Then, $K\left(x\right)=2,\forall x\in {\mathbb{R}}^{3}\mathrm{ s}.\mathrm{t}. \underset{y\in C}{\mathrm{min}}\left(\left|x-y\right|\right)>r$ is set. In addition, $r$ is empirically set to 10 mm, and${S}_{2} :=\{x\in {\mathbb{R}}^{3} :K(x)=2\}$. Therefore, in the current image$K$, any pixel with a value of 1 is located inside the lumen, and any pixel with a value of 2 is outside the lumen. The zero-valued pixels ${S}_{0} :=\{x\in {\mathbb{R}}^{3} :K(x)=0\}$ remain undetermined. To find the categories of undetermined pixels, a distance-modulated shortest-path algorithm is employed (see Algorithm 1). Accordingly, for$p\in I$, denote as $N(p)$ the Moore neighborhood of$p$, i.e., the 8 directly neighboring pixels of $p$ in 2D and 26 voxels of $p$ in 3D. The influence of a point $q\in N(p)$ on $p$ is defined as${\Vert I(q)-I(p)\Vert }_{2}\in [\mathrm{0,1}]$, where $I(p)$ is the intensity at point$p$. From Algorithm 1, it can be seen that$\forall p\in I$, $K(p)$ under a steady state is determined by$K({l}^{*})$:

3 $${l}^{*}=\underset{l\in S}{\mathrm{argmin}}\underset{{q}_{i}\in H(l,p)}{\mathrm{min}}{\{\Vert I({q}_{1})-I(l)\Vert }_{2}+{\Vert I({q}_{2})-I({q}_{1})\Vert }_{2}+\cdots +{\Vert I(p)-I({q}_{n})\Vert }_{2}\},$$

where $S$ is the set of seed pixels, and $H$($l,p$) is any path connecting pixel $p$ and seed $l$. Note that the inner part of Eq. (3) can be considered as the shortest weighted distance from $p$ to $l$, and the outer part is the clustering based on this distance. As an advantage of this formulation, Eq. (3) can be solved efficiently using Dijkstra’ s algorithm [37].


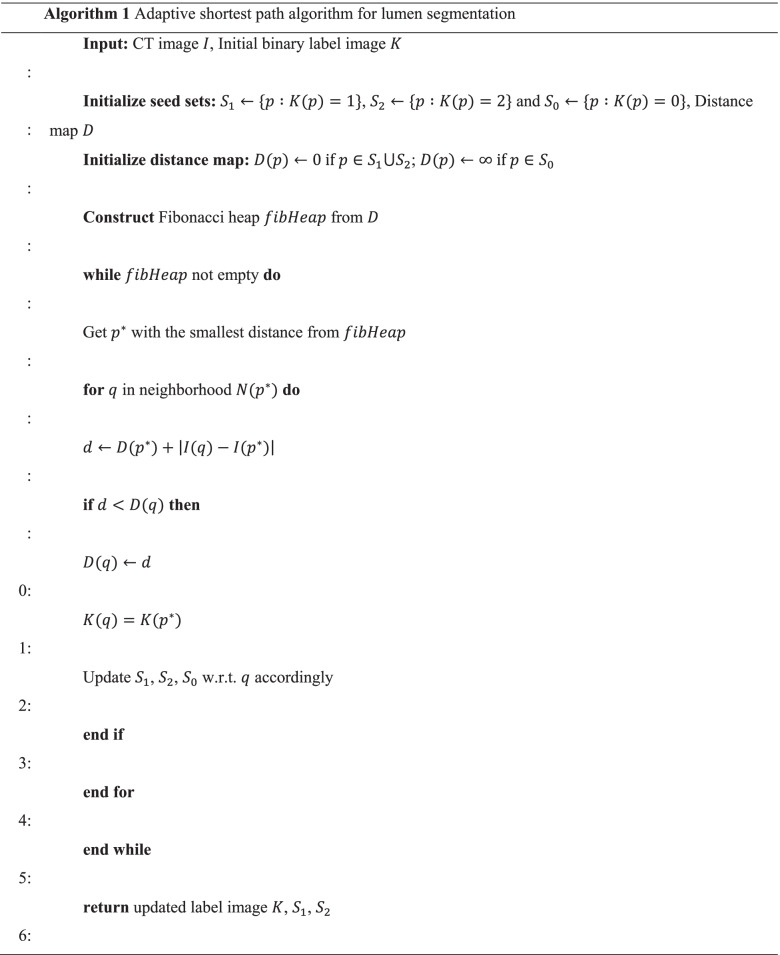


Conceptually, for each point $p$ in the undetermined group, two short curves were plotted. One curve was connected to the seeds using label-1 and the other was linked to the seeds using label-2. However, the lengths of the two curves were not defined in a Euclidean sense. More explicitly, if the curve connects two neighboring pixels, $x$ and $y$, then, the local length is $\left|I\left(x\right)-I\left(y\right)\right|$. Based on this definition, the length of a curve not only represents the spatial distance from the undetermined region to the seeded region, it also signifies the similarity between the two regions. Therefore, when starting from an undetermined pixel, if the shortest curve connecting to any seeds with value-1 has a length smaller than that of seeds with value-2, then this undetermined pixel should belong to 1-group. Accordingly, each undetermined pixel is computed. All pixels have either value-1 or value-2 at the end of the computation, where value-1 pixels cover the region of the vessel lumen. Therefore, the final segmentation provided the extracted vessel lumen, which could be used for further analysis.

### Implementation and user interface design

**Fig. 2:**
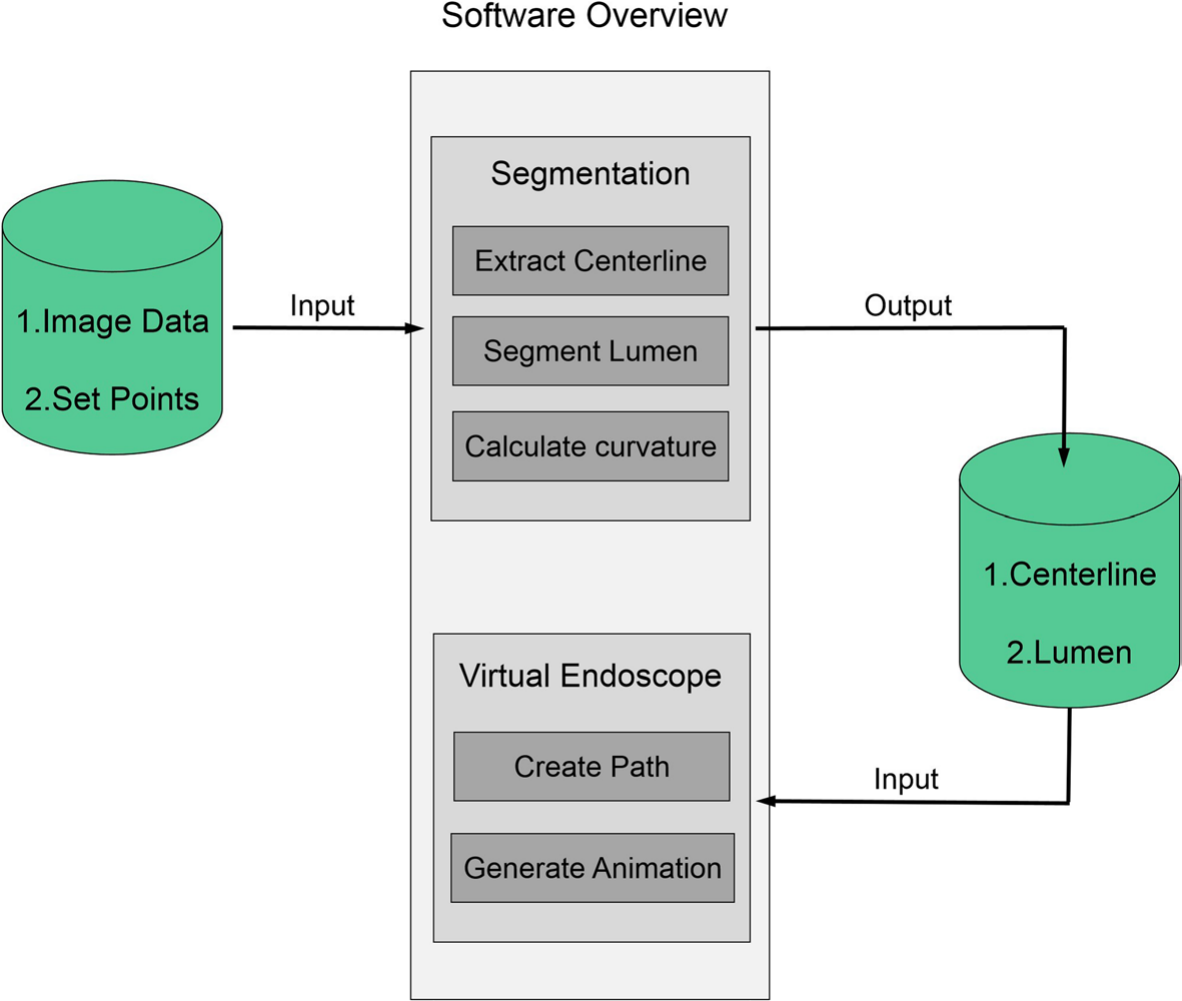
Software overview. After inputting the 3D CT image and setting the points, the software will output the segmented lumen and generate a VE animation, allowing a statistical analysis to be conducted

Figure 2 illustrates the end-to-end infrastructure of the developed software. It allows flexible user interactions, generates centerline extraction and lumen segmentation tasks in the backend, and then produces results with a virtual endoscope.

Because the proposed algorithm includes different user interactions, a well-designed graphical user interface (GUI) is critical. As one of the leading open-source medical platforms, 3D Slicer is an open-source software platform for medical image informatics, image processing, and 3D visualization. It is also noteworthy that 3D Slicer supports three types of modules: a command line interface, loadable modules, and scripted modules [38].

**Fig. 3:**
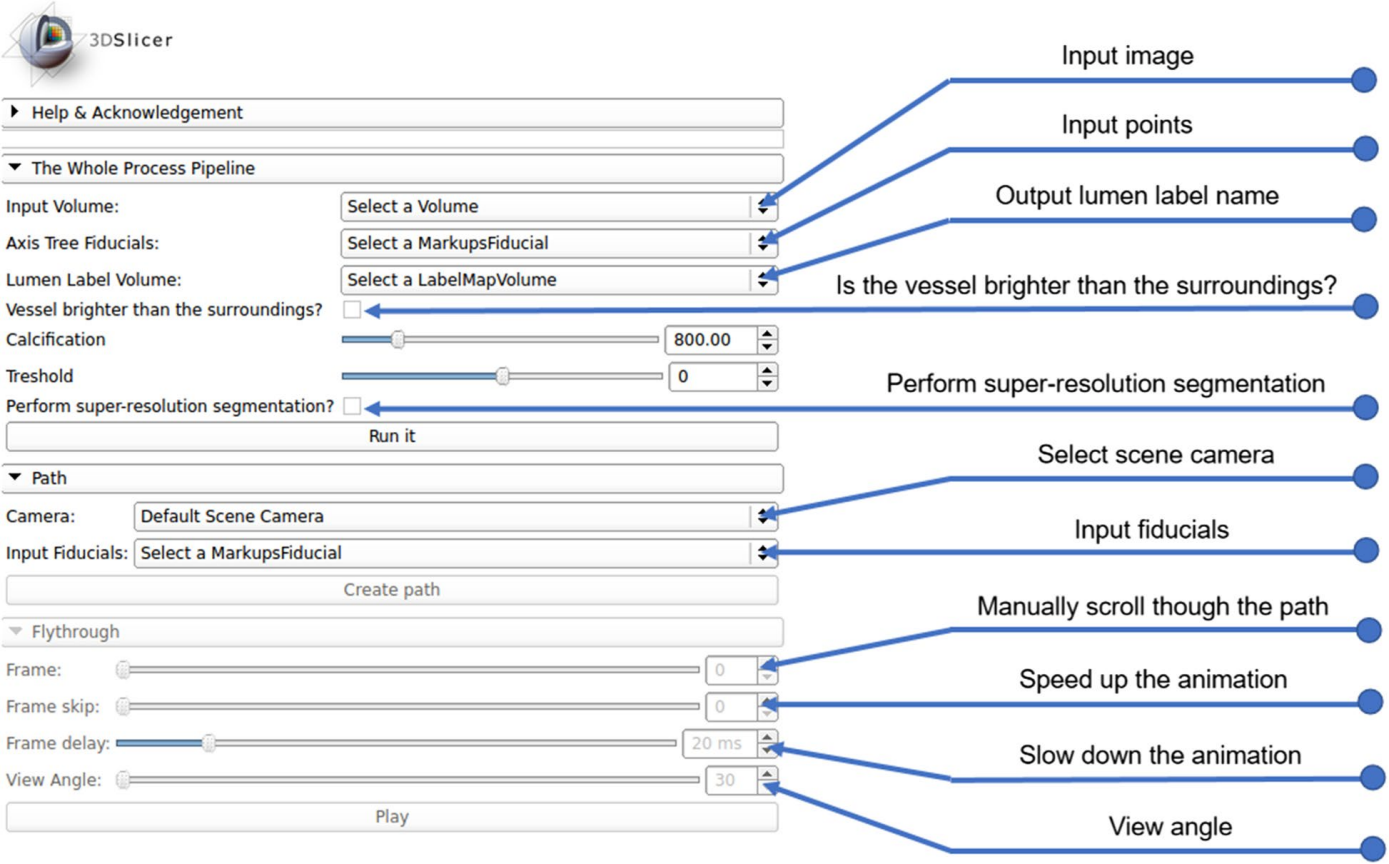
UI of the proposed software

In the present study, because the interactive editor tool of 3D Slicer is utilized, which contains a variety of interactive segmentation effects, a Python scripting interface was used. The UI of the module is shown in Fig. 3.

## Results

### Centerline extraction

**Fig. 4:**
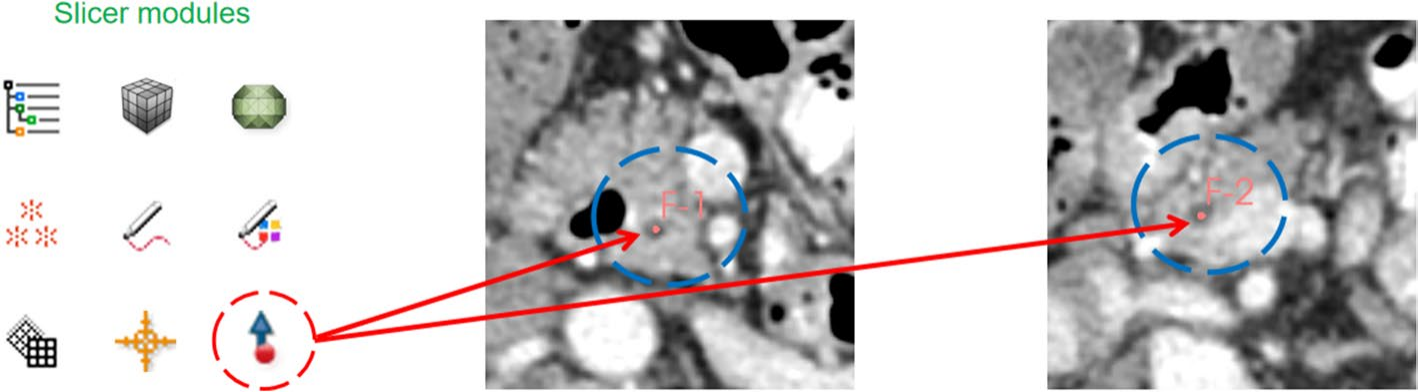
Use of the Fiducial marker tool to pin-point an area on the slices. From left to right: Slicer modules and the head and tail of the pancreatic duct. The red circle indicates the Fiducial marker tool, and the blue circles indicate the points (point F-1 is at the head of the duct and F-2 is at the tail)

To extract the duct centerline, a user needs to place one fiducial point at the beginning and end of the duct. As shown in Fig. 4, this can be achieved using the Fiducial module of Slicer. The information is transmitted to the preprocessing modules of the virtual endoscope system.

**Fig. 5:**
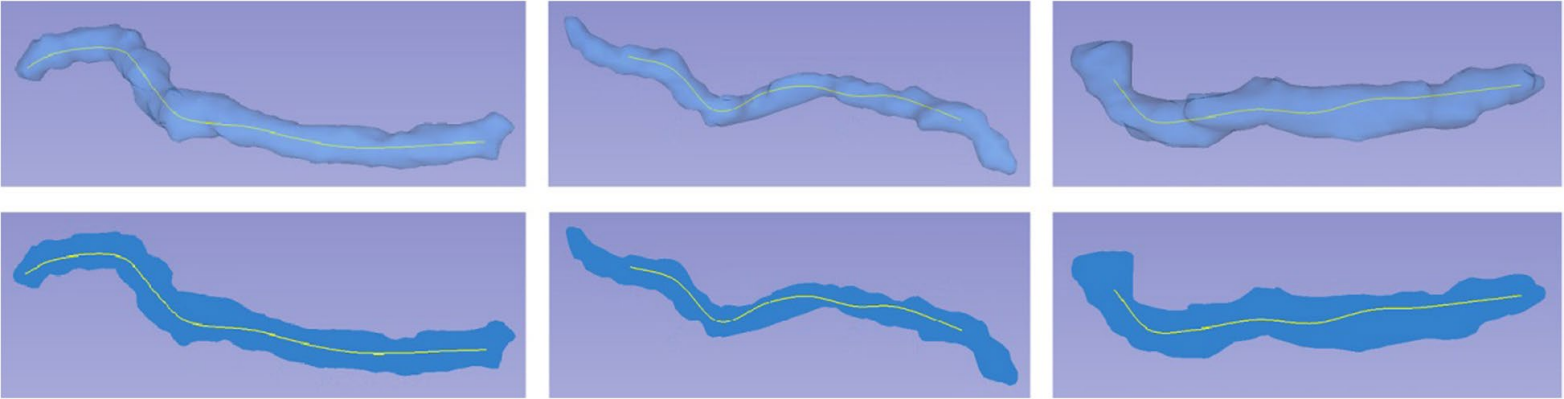
Results of centerline extraction, in which the first row represents the 3D model and the second row denotes the network topology

Consequently, the centerline of the pancreatic duct was computed. The centerline is illustrated in a 3D model in the first row of Fig. 5, and the network topology is displayed in the second row.

### Lumen segmentation

Subsequently, the endoscopy module was built to conduct a fly-through visualization of the duct. More explicitly, a spline curve was constructed from the centerline. The virtual camera was then positioned along the spline curve with its orientation pointing in the tangential direction of the curve. Subsequently, the camera was moved along the curve. It is obvious that, in addition to a centerline extraction, an accurate lumen segmentation is critical for visualization.

**Fig. 6:**
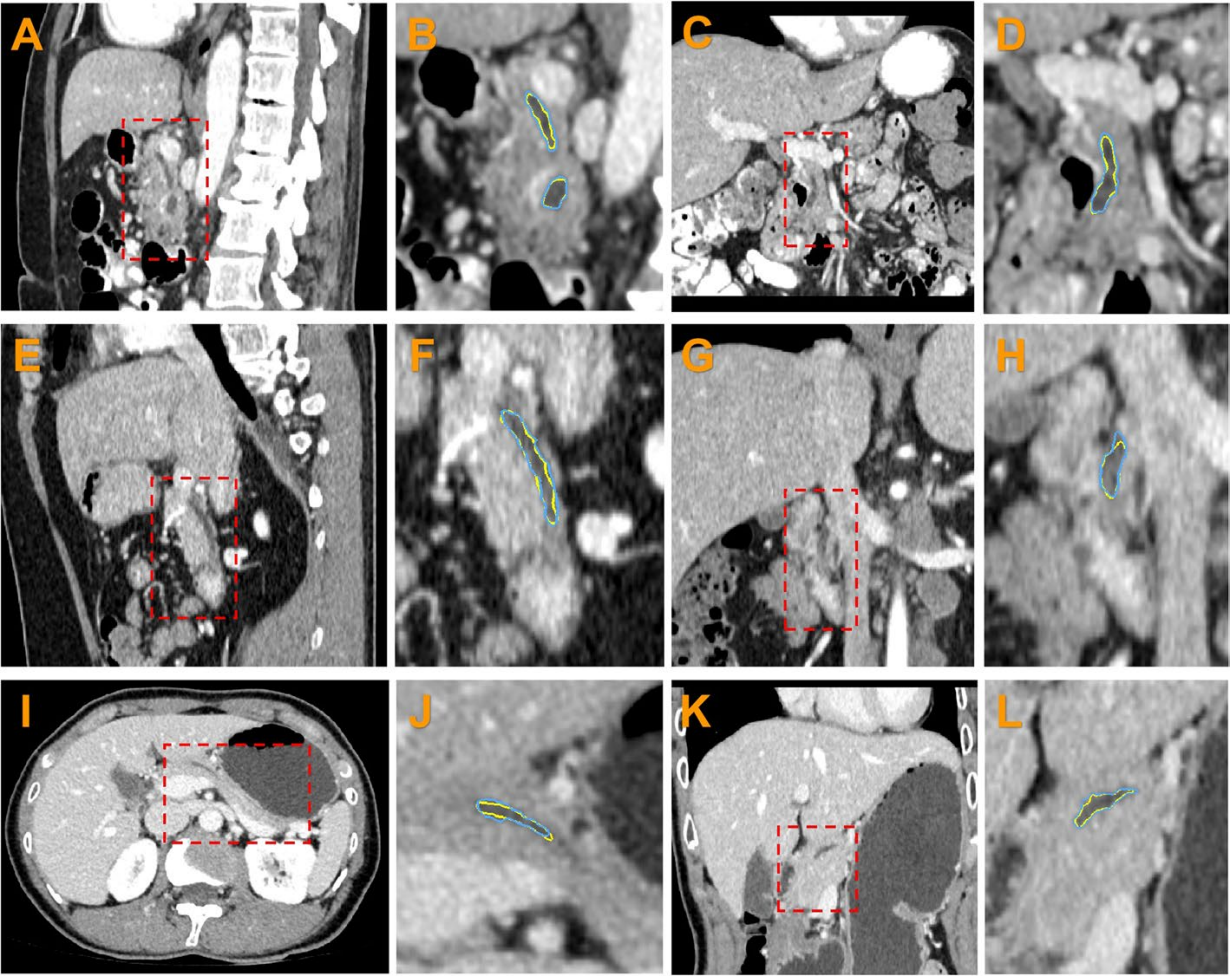
Segmentation results shown in 2D views: **a**, **c**, **e**, **g**, **i**, and **k** are the original images, and **b**, **d**, **f**, **h**, **j** and **l** show the magnified views of the segmentation contour. The results of the proposed method are marked in blue, and those of the manual segmentation are marked in yellow

**Fig. 7:**
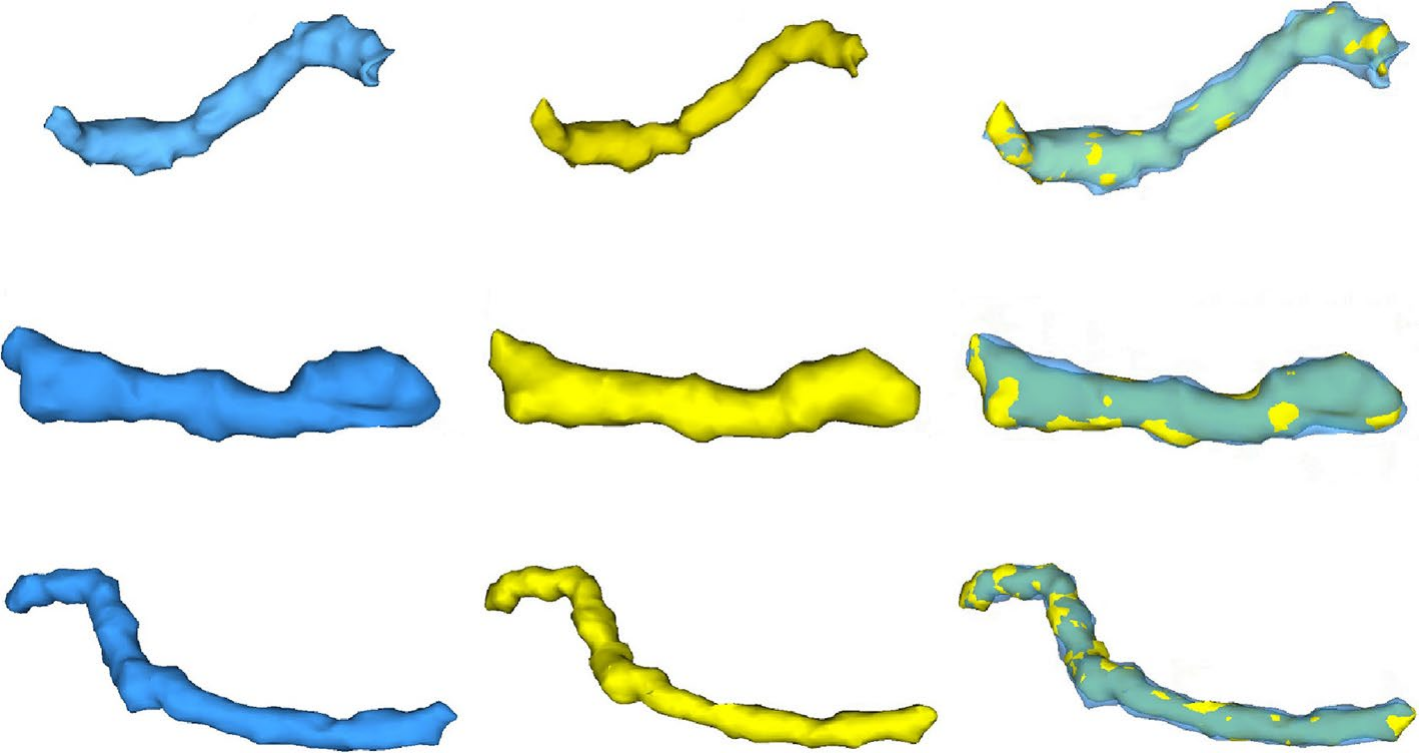
Segmentation results shown in Fig. 6 displayed in a 3D view. The Dice coefficients of the first, second, and third row examples are 0.90, 0.87, and 0.84, respectively

The proposed method was compared with a manual segmentation. In this test, a CT image obtained from NIH-TCIA was used [39]. The dataset contained 82 abdominal contrast-enhanced 3D CT images. The CT scans have pixel resolutions of 512 × 512 with varying pixel sizes and slice thicknesses of between 1.5 and 2.5 mm, acquired on Philips and Siemens MDCT scanners (120 kVp tube voltage). Manual segmentation was applied by a physician. Twenty cases of pancreatic ducts that could be distinguished by the human eye were marked as the testing data. In Fig. 6, six examples of the segmentation results from the proposed method and a manual segmentation are presented. Examples of the same lines are from the same person. The segmentation results of the proposed and manual segmentation methods are marked in blue and yellow, respectively. Figure 7 shows the 3D rendering of these examples. The Dice coefficients of the first, second, and third row examples were 0.90, 0.87, and 0.84, respectively. The average Dice coefficient across the entire testing dataset was 0.85, and the standard deviation was 0.04. This accuracy is higher than that of the most recent pancreatic duct segmentation approach [27]. In addition, as one important factor, the developed framework supports efficient user interactions to provide endpoints for the duct.

### Fly-through visualization

**Fig. 8:**
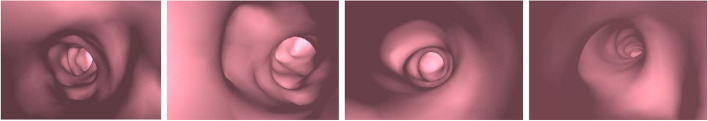
Results of simulated fly-through of the lumen

Once the lumen surface was reconstructed, the simulated fly-through of the lumen was achieved. Figure 8 shows four cases of a lumen fly-through. It can be seen that the inner surface is quite smooth without nodules/cysts because these subjects were all healthy. Further research will be conducted to effectively promote software that can be used by clinicians, thus enabling the proposed VP to be validated through optical pancreatoscopy.

### Curvature visualization

**Fig. 9:**
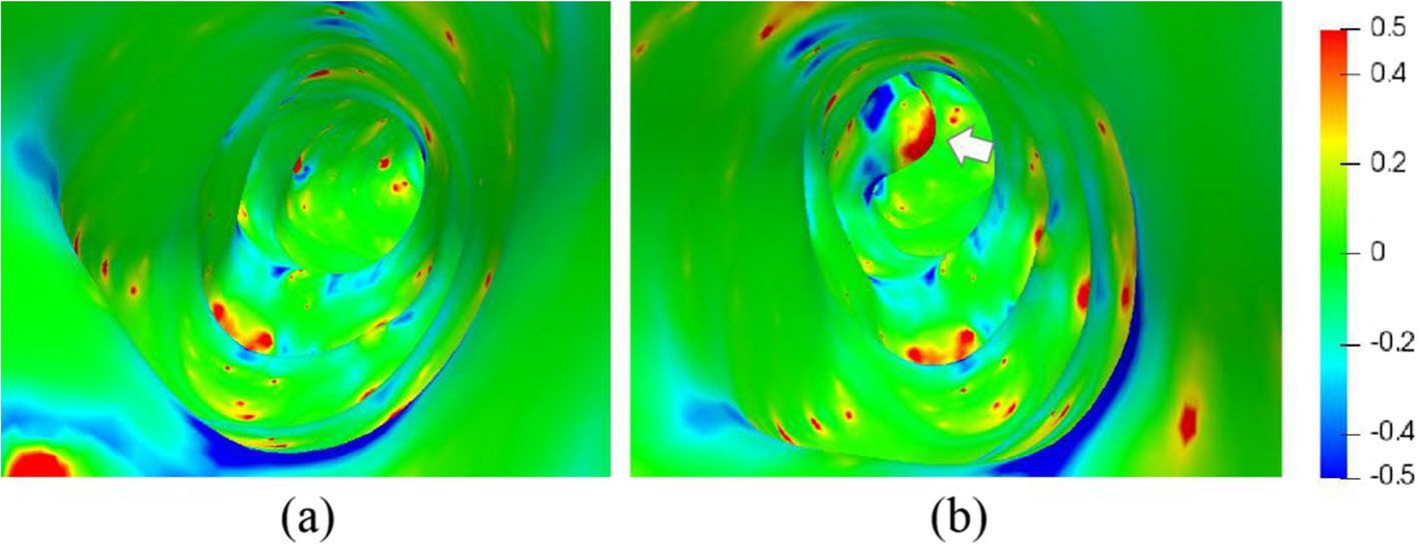
Results of curvature of the lumen. **a** Pancreatic duct of healthy subject; **b** Pancreatic duct with a simulated cyst (white arrow)

Nodules in the lumen duct have a characteristically high Gaussian curvature on their surfaces. To better visualize the nodule, the proposed software calculated and visualized the curvature of the pancreatic duct. The magnitude of the curvature is mapped into the color space as a “curvature map.” Figure 9a shows a curvature map of a healthy human pancreatic duct. The green color indicates regions with a small curvature, whereas the red and blue regions have high positive and negative curvatures, respectively. On the lumen surfaces of the healthy individuals, only sporadic regions with high curvatures (absolute values) can be observed.

The investigation of patients with nodules is ongoing. However, to demonstrate the capability of the proposed platform, one nodule was artificially generated on the lumen surface and a curvature map was created. Figure 9b shows the curvature of the same pancreatic duct in Fig. 9a, but with a simulated cyst in the inner surface. It can be seen that the cyst part has a prominent bright red color.

### User study

To conduct the software testing, five MS students with biomedical backgrounds were trained. The students used VP for interactive segmentation of pancreatic ducts and Slicer for a manual segmentation. A total of three pancreatic ducts were segmented per person, and the average time consumption and average Dice coefficients of the segmentation results for different methods were then recorded. Table 1 presents the test results. According to these results, VP is markedly faster (approximately 3.7 × faster than Slicer for segmentation of the pancreatic ducts), while maintaining a high approximation accuracy.

**Table 1:** Comparison of pancreatic duct segmentation using VP and Slicer

**Software**	**Time (s)**	**Dice coefficients**
Slicer	664.67 $\pm$ 81.5	0.928 $\pm$ 0.026
VP	181.67 $\pm$ 16.86	0.871 $\pm$ 0.008

## Discussion and conclusions

In this study, an open-source software program was developed for VP. Specifically, 3D pancreatic duct segmentation, lumen reconstruction, visualization of the VP, and curvature of the pancreatic duct were demonstrated. The user only needs to mark the head and tail of the pancreatic duct with a single point each, and the segmentation algorithm is then automatically applied. The average time for the proposed software to segment a single pancreatic duct after a user click is less than 1 s (on a computer with a 3.4-GHz CPU and 8 GB of RAM). Compared with an approximately 10-min manual segmentation, this is a significant improvement in efficiency. The segmentation results of the proposed method and manual segmentation were presented and compared. The proposed method can overcome certain drawbacks of traditional endoscopy, such as invasiveness and possible complications after examination. Such a virtual procedure can be applied prior to the application of optical pancreatoscopy, allowing an initial assessment to be conducted and the necessity of traditional pancreatoscopy to be evaluated.

Moreover, this software was popularized and made freely available for validation, testing, and utilization. Ongoing research will include determining its effectiveness with respect to traditional pancreatoscopy and evaluating its diagnostic capability for diseases such as cysts and IPMNs.

## Data Availability

The present manuscript reports an open-sourced software package and the data used in this manuscript were from the TCIA Pancreas-CT dataset.

## Abbreviations

VP: Virtual pancreatoscopy
CT: Computed tomography
IPMN: Intraductal papillary mucinous neoplasm
VE: Virtual endoscopy
VC: Virtual colonoscopy
CTC: Computed tomography colonography
OC: Optical colonoscopy
GUI: Graphical user interface
